# Diabetes and urbanization in the Omani population: an analysis of national survey data

**DOI:** 10.1186/1478-7954-4-5

**Published:** 2006-04-24

**Authors:** Siba Al-Moosa, Sara Allin, Nadia Jemiai, Jawad Al-Lawati, Elias Mossialos

**Affiliations:** 1 The London School of Economics and Political Science, LSE Health and Social Care, UK; 2 Ministry of Health, Oman

## Abstract

**Background:**

The prevalence of type 2 diabetes in Oman is high and appears to be rising. Rising rates of diabetes and associated risk factors have been observed in populations undergoing epidemiological transition and urbanization. A previous study in Oman indicated that urban-dwellers were not significantly more likely to have diabetes. This study was undertaken to determine if a more accurate urban and rural categorization would reveal different findings.

**Methods:**

This study included 7179 individuals aged 20 years or above who participated in a cross-sectional interviewer-administered survey in Oman including blood and anthropomorphic tests. Multiple logistic regression analyses were conducted to analyze the factors associated with diabetes, first in the whole population and then stratified according to region.

**Results:**

The prevalence of diabetes (fasting blood glucose ≥ 7 mmol/l) in the capital region of Muscat was 17.7% compared to 10.5% in rural areas. The prevalence of self-reported diabetes was 4.3%. Urban residence was significantly associated with diabetes (adjusted odds ratio (OR) = 1.7, 95% confidence interval (CI): 1.4–2.1), as was age (OR = 1.2, 95% CI: 1.1- 1.2), obesity (abnormal waist circumference) (OR = 1.8, 95% CI: 1.5–2.1), and systolic blood pressure (SBP) 120–139 (OR = 1.4, 95% CI:1.04–1.8), SBP 140–159 (OR = 1.9, 95% CI: 1.4–2.6), SBP ≥ 160 (OR = 1.7, 95% CI: 1.2–2.5). Stratified analyses revealed higher education was associated with reduced likelihood of diabetes in rural areas (OR = 0.6, 95% CI: 0.4–0.9).

**Conclusion:**

A high prevalence of diabetes, obesity, hypertension and high cholesterol exist in the Omani population, particularly among urban-dwellers and older individuals. It is vital to continue monitoring chronic disease in Oman and to direct public health policy towards preventing an epidemic.

## Background

Epidemiological studies reveal rising rates of type 2 diabetes worldwide, notably in developing countries undergoing epidemiological transition from communicable to chronic diseases [1,2]. This has also been observed in certain populations that have undergone relatively rapid transition from rural to urban lifestyle [3-5]. Studies in low- and middle-income countries have identified that risk factors for chronic diseases are more prevalent in urban than rural areas [6-9].

Although the Sultanate of Oman has been successful in reducing communicable diseases and increasing life expectancy at birth to 76 years for women and 71 for men [10], rapid cultural changes and social advances since 1970 have led to the manifestation of a wide range of non-communicable diseases [11]. The 2004 population is extremely young, with only 3% aged 60 years or older [10].

The fifth Health Development Plan in Oman (1996–2000) identified, for the first time, diabetes as a major concern and set specific goals to prevent further progression of the disease [12]. The National Diabetes Survey conducted in 1991 showed that 10% of the Omani population over the age of 20 years had diabetes mellitus, resulting in an estimated 80,000 people living with diabetes [13]. Also, a recent study of metabolic syndrome in an Omani city, Nizwa, reported a prevalence of high fasting glucose of 18% in 2001 [14].

A recent study conducted in Oman [11] identified that diabetes was associated with the classic risk factors [15], but not urbanization. The authors' definition of urbanization was based on each wilayate (district) being divided into two sections: the first section comprised the wilayates' centers representing the urban area and the second section comprised the villages or remote parts, which represent the rural areas [11]. According to this definition, 73.1% of the population resides in urban areas.

Consistent with this definition, the World Bank and UNDP also present high figures of urbanization in Oman. Specifically, in 2001 an estimated 76% of the population was considered urban [16], compared to 77% in 2002 [17].

However, other studies suggest a very different urban-rural distribution (e.g. 11% urban in 1990 [18]; 10.6% urban in 1990 and 15.1% in 2000 [19]). Similarly, we believe the only area in Oman that can be considered urban is the capital region of Muscat, constituting 21.7% of the population in 2000.

The population density (persons/km^2^) in the region of Muscat is 162.1, compared to 52.3 in the second most populated region (Al Batinah), with the remaining regions relatively sparsely populated (0.3–15.8 persons/km^2^) [20]. In addition, about half of all commercial banks, vehicles on the road, electricity connections, and telephone lines in Oman are found in the capital region of Muscat, which can be seen as an indirect indication of urbanization [20]. Furthermore, health facilities are concentrated in the capital region; about 30% of all hospital beds, and 40% of private health clinics are located in Muscat [20]. Moreover, the average occupancy rate of the Muscat hospitals is much higher than the other regions: 66% compared to an average of 46% across the remaining regions [20]. The only international airport in the country is located in Muscat; the same is true for the only government university. Finally, the presence of American companies further demonstrates the potentially different lifestyle among those living in Muscat; there are four Starbucks and four McDonalds restaurants in Oman, all of which reside in Muscat.

Socio-demographic characteristics of the urban and rural populations also differ significantly. Table 1 shows the age, sex and educational distribution of the populations in Muscat and the remaining regions. Overall, Muscat has a younger, better educated population with a slightly smaller average family size.

**Table 1:** Socio-demographic characteristics of urban (Muscat) and rural populations

	**Urban (%)**	**Rural (%)**
**Age**		
20–29	40.5	41.2
30–39	24.3	20.6
40–49	14.5	10.9
50–59	10.7	13.9
≥ 60	10.0	13.3
**Sex**		
Male	48.8	50.3
Female	51.2	49.7
**Education level**		
Illiterate	24.8	36.1
Less than secondary	37.5	41.8
Secondary or above	37.7	22.1
**Average family size**	7.3	8.0

Note: Data are from the 2000 National Health Survey

The objectives of the present study are twofold: to measure the prevalence of diabetes and associated coronary risk factors across demographic and socioeconomic descriptors; and to determine the significant factors associated with diabetes, in particular to examine whether urban residence as we have described above (i.e. living in the capital region) is independently associated with diabetes, adjusting for other risk factors and demographic factors.

## Methods

### Study population and sampling method

The sample for the 2000 National Health Survey was chosen so that it would represent the ten regions in the Sultanate. First, a multi-stage stratified probability-sampling design was used to randomly select a number of wilayates (districts) from each region, according to proportional allocation of population size in each region. There are 59 wilayates in Oman, of which 16 were chosen for the survey. Second, within the wilayates, enumeration areas (as defined by the 1993 census) were randomly selected. Third, within the enumeration areas, households were randomly selected. The present analysis considered the capital region of Muscat to be urban, and considered the remaining nine regions to be rural.

All subjects aged 20 and above in each of the chosen households were requested to participate in the survey. Out of the 2044 occupied households in the sample, 1968 were interviewed with a total of 17,574 subjects (96%), of which 7179 were aged 20 or older. The response rate of subjects aged 20 or above was relatively high for all medical measurements: height, weight and blood pressure (90%), waist measurement (89%), hip measurement (86%), fasting blood glucose (81%) and serum cholesterol samples (82%).

### Data collection

A questionnaire developed by the Ministry of Health was administered by trained interviewers. The questionnaire included demographic background (age, sex, marital status, educational status, and working status) and self-reported diabetes mellitus, hypertension and smoking habits. The survey protocol also included measurements of blood pressure, weight, height, waist and hip circumference, taken by nurses according to WHO recommendations [21]. Blood samples of venous fasting blood glucose and serum cholesterol were also collected. Details of the sample collections are given elsewhere [22]. Fasting was ensured through visits by the interview teams to the houses on two consecutive days: on the first day, participants were requested to fast after dinner; on the second day the participants were visited in the morning, and blood samples were taken only for those individuals who confirmed they had fasted.

Diabetes was determined through adding the individuals with self-reported diabetes to those with a fasting blood glucose of ≥ 7.0 mmol/l, following the WHO criteria for diagnosis of glucose intolerance [21].

### Data analysis

Blood pressure was measured twice for each individual and mean systolic blood pressure (SBP) in mmHg was separated into four categories: <120; 120–139; 140–159; and ≥ 160. Hypertension in this study was defined as SBP ≥ 140 mmHg. Cholesterol levels (mmol/L) were divided into quartiles (<4.3; 4.3–5; 5.1–5.8; and >5.8); cholesterol ≥ 5 mmol/L was considered to be high.

Measures of obesity included waist circumference, body mass index [BMI; weight in kilograms divided by the square of the height in meters (kg/m^2^)], and waist-to-hip ratio. Following WHO guidelines, waist circumference was considered to be abnormal ≥ 102 cm for men and ≥ 88 cm for women; and waist-to-hip ratio (WHR) was considered to be abnormal >0.95 cm for men and >0.85 cm for women. Respondents were classified as overweight if BMI ≥ 25 and <30 and obese if BMI ≥ 30.

The demographic variables include: age, grouped into 17 five-year age bands between 20 and 109 years; sex; and marital status, measured as married or not married (including divorced, separated, widowed or never married). Whether the individual smoked was also included. Level of education is separated into three groups: illiterate; less than secondary school; and secondary school and above. No other available measure of income or socioeconomic status was available in this survey.

Place of residence was used to categorize individuals into urban and rural. Those living in Muscat were considered to be urban-dwellers, while those residing in the rest of the country were considered to be living in rural areas.

The prevalence of diabetes, hypertension, high cholesterol, and obesity by region, demographic and educational status was measured. Then multiple logistic regression analyses were conducted to analyze the significant factors associated with the dependent variable, diabetes, first in the whole population and then stratified according to region. Independent variables were selected on the basis of the literature of diabetes risk factors [23]. Due to the high degree of colinearity between the three measures of obesity, only waist circumference was chosen for the regression models, as recommended by WHO. Most of the variables included within the models are categorical, thus dummy variables are created. Results are presented as crude and adjusted odds ratios with associated 95% confidence intervals. All analyses were conducted using Stata 8; *p*-value of <0.05 was considered significant.

## Results

Table 2 presents the prevalence of diabetes, hypertension, high cholesterol and obesity across the identified independent variables. Among the population aged 20 or above, the overall prevalence of diabetes was 11.6% and varied according to urban or rural residence, age, marital status, educational level, smoking status, measures of obesity (BMI, WHR, waist circumference), cholesterol and SBP. The prevalence of hypertension (mean SBP ≥ 140 mmHg), and obesity (BMI ≥ 30) among the adult population were 21.5% and 19.1% respectively. High cholesterol (≥ 5 mmol/L) affects more than half of the adult Omani population.

**Table 2:** Prevalence (%) of diabetes, hypertension, high cholesterol, and obesity by urban residence and selected variables.

	**Diabetes **(n = 676/5840)	**Hypertension **(n = 1389/6464)	**Cholesterol**=**5 **(n = 2974/5876)	**BMI**=**30 **(n = 1224/6430)
Total	11.6	21.5	50.6	19.1
**Residence**				
Urban	17.7	26.4	50.0	19.9
Rural	10.5	20.2	50.7	18.8
**Age**				
20–29	3.6	7.95	32.1	13.4
30–39	8.5	14.5	55.7	24.8
40–49	17.1	25.7	59.4	28.6
50–59	22.6	39.3	69.2	22.2
≥ 60	23.1	52.2	71.3	15.0
**Sex**				
Male	11.8	32.5	50.8	15.5
Female	11.3	22.7	50.4	22.2
**Marital status**				
Married	13.2	22.8	55.8	21.6
Not married	8.0	18.4	39.3	13.5
**Education level**				
Illiterate	17.1	34.7	62.1	19.8
Less than secondary	10.2	16.9	48.6	20.1
Secondary or above	5.4	9.9	36.8	16.1
**Smoker**				
Yes	12.2	25.2	50.3	14.5
No	11.5	21.2	50.6	19.4
**BMI (kg/m**^2^**)**				
<18.5	6.9	14.7	36.4	-
18.5–24.99	8.1	18.5	44.8	-
25–29.99	14.7	22.6	58.0	-
≥ 30	16.5	29.7	58.9	-
**WHR (cm)**				
Normal	8.2	16.4	43.9	12.3
Abnormal (men >0.95; women >85)	15.5	27.3	58.4	26.6
**Waist circumference (cm)**				
Normal	9.5	18.5	47.2	7.8
Abnormal (men = 102; women = 88)	18.1	30.7	61.5	53.2
**Cholesterol (mmol/L)**				
<4.3	5.5	13.2	-	12.8
4.3–5	8.0	18.5	-	18.2
5–5.8	12.6	22.8	-	19.8
≥ 5.8	17.8	32.9	-	23.5
**SBP (mmHg)**				
<120	6.2	-	44.2	12.9
120–139	9.8	-	47.8	18.8
140–159	19.6	-	60.9	25.3
≥ 160	24.4	-	74.5	28.9

Note: SBP is systolic blood pressure; BMI is body mass index; WHR is waist-to-hip ratio

Furthermore, diabetes affects a much greater proportion of the urban population (17.7%) than rural (10.5%). Similarly, hypertension is more common in urban (26.4%) than in rural areas (20.2%). The difference in prevalence of obesity in urban areas and rural areas appears to be less significant, as with the prevalence of high cholesterol. A gradient in unstandardized diabetes prevalence was also observed with education suggesting that the higher educated individuals were less likely to have diabetes than the illiterate or less educated groups.

Table 3 shows the variables that are significantly associated with diabetes, ceteris paribus. Of the 7179 respondents aged 20 or over, 5847 individuals were included in the analysis due to missing data for one or more of the variables. Individuals living in the urban capital region were almost twice as likely to have diabetes as those living in the rural areas. Furthermore, advanced age, being married, and abnormal waist circumference also increased the odds of diabetes.

**Table 3:** Significant factors associated with diabetes (p < 0.05)

	**No diabetes **(n = 5164)	**Diabetes **(n = 676)	**Crude OR (95% CI)**	Adjusted OR *(95% CI)
	n (%)	n (%)		

**Residence**				
Urban	741 (82.3)	159 (17.7)	1.8 (1.5–2.2)	1.7 (1.4 – 2.1)
Rural	4430 (89.6)	517 (10.5)	-	-
**Age**^±^			1.2 (1.2–1.3)	1.2 (1.1- 1.2)
**Marital status**				
Not married	1721 (92.0)	149 (8.0)	-	-
Married	3431 (86.8)	523 (13.2)	1.8 (1.5–2.1)	1.4 (1.1–1.7)
**Waist circumference (cm)**				
Normal	4023 (90.5)	422 (9.5)	-	-
Abnormal	1148 (81.9)	254 (18.1)	2.1 (1.8–2.5)	1.8 (1.5- 2.1)
**SBP (mmHg)**				
<120	1231 (93.8)	81 (6.2)	-	-
120–139	2871 (90.2)	311 (9.8)	1.6 (1.3–2.1)	1.4 (1.04 – 1.8)
140–159	751 (80.4)	183 (19.6)	3.7 (2.8–4.9)	1.9 (1.4 – 2.6)
≥ 160	264 (75.6)	85 (24.4)	4.9 (3.5–6.8)	1.7 (1.2 – 2.5)

Note: SBP is systolic blood pressure. * Odds ratios adjusted for all variables included in the model. ^± ^Age is grouped into 17 five-year age bands between 20 and 109 years. Therefore for every five year increase in age, there is a 1.2 greater odds of having diabetes.

A strong gradient was apparent with SBP: compared to SB p < 120 mmHg the adjusted OR for diabetes increased from 1.4 (SBP: 120–139 mmHg) to 1.9 (SBP: 140–159 mmHg) and decreased to 1.7 (SBP = 160 mmHg). The lack of a linear relationship between SBP and diabetes is likely a result of the small sample of individuals in the highest SBP group.

After controlling for the other factors, education was not significantly associated with diabetes, except with stratified analyses by region (see Table 4). These stratified analyses revealed that higher educated individuals, particularly in rural areas, were less likely than illiterate individuals to have diabetes.

**Table 4:** Significant factors associated with diabetes in urban and rural areas (p < 0.05) or (p < 0.10)*

	**Diabetes in urban areas **(n = 159)		**Diabetes in rural areas **(n = 517)	
	n (%)	Adjusted OR (95% CI)	n (%)	Adjusted OR (95% CI)

**Age **^±^	-	1.2 (1.1–1.2)		1.2 (1.17–1.24)
**Marital status**				
Not married	35 (11.1)	-	114 (7.3)	-
Married	120 (20.7)	1.6 (1.0–2.3)	403 (11.9)	1.3 (1.0–1.7)
**Educational level**				
Illiterate	68 (28.0)	-	323 (15.6)	-
Less than secondary	43 (12.9)	0.6 (0.4–1.0)*	196 (9.8)	1.3 (1.0–1.6)
Secondary or above	43 (14.0)	1.0 (0.6–1.7)	32 (3.0)	0.6 (0.4–0.9)
**Waist circumference (cm)**				
Normal	99 (14.7)	-	323 (8.6)	
Abnormal	60 (26.7)	1.5 (1.0–2.3)	194 (16.5)	1.9 (1.5–2.3)
**SBP (mmHg)**				
<120	15 (8.4)		66 (5.8)	-
120–139	59 (13.3)	1.2 (0.7–2.3)	252 (9.2)	1.4 (1.0–1.8)
140–159	51 (28.2)	2.7 (1.4–5.1)	132 (17.5)	1.7 (1.2–2.4)
≥ 160	28 (32.6)	2.2 (1.0–4.7)	57 (21.7)	1.6 (1.1–2.5)

Note: SBP is systolic blood pressure

* Odds ratios adjusted for all variables included in the model.

^± ^Age is grouped into 17 five-year age bands between 20 and 109 years.

## Discussion

As expected with the use of a more accurate categorization of urban and rural areas in Oman, residence was found to be a significant independent factor for diabetes similar to other epidemiological studies [24,25]. Because the impact of urban residence on likelihood of diabetes persisted after adjusting for demographic and educational variables, there are some unobserved unique features of urban populations that increase their risk of diabetes. These variables are likely to relate to lifestyle factors or perhaps to characteristics of employment.

Possibly explaining the higher prevalence of diabetes in the urban area is the change in dietary patterns, physical activity patterns and lifestyle associated with urbanization (which has been taking place to a large extent in the past 30 years, as witnessed by the increasing migration of Omanis to Muscat) [26,27]. Moreover, in the past 30 years Oman experienced rapid socioeconomic developments that were associated with a sharp rise in car ownership, an increase in consumption of high fat caloric-dense food, refined sugar and salt, and increased rates of smoking [28].

It is unlikely that the elevated rate of diabetes in the capital region is due to lack of awareness or diagnosis among rural-dwellers, since the same haematological criteria to determine diabetes were used for all individuals. Because of the lack of available information on income, lifestyle factors such as diet and exercise, household expenditure, or socioeconomic status, it is not possible to test whether these factors may help explain the relationship between urbanization and diabetes.

The results of this study indicate that hypertension is significantly associated with diabetes, particularly among urban-dwellers. Also, the prevalence of hypertension is higher in the capital region than the rural areas. These findings are consistent with previous studies [8,13,29-32], and indicate that individuals in the capital region are at higher risk for developing both diabetes and cardiovascular disease, due to elevated prevalence of coronary risk factors, than those in rural regions of Oman.

Almost one-fifth of the adult Omani population are obese. The past decade has witnessed an increase in the rate of obesity and overweight among Omani men, while a declining trend has been observed among Omani women [28]. In the urban area, this high prevalence could be attributed to rural to urban migration after the 1980s oil boom [28]. Although higher than among men, the decreasing rate of obesity among women has been explained by increasing educational levels, declining fertility rates and improved awareness of self-image [28].

Crude estimates indicate that illiterate and less educated individuals are more likely to suffer from diabetes, high blood pressures and high cholesterol. It appears that when all the other factors are considered, education is not independently associated with diabetes. However, when separate analyses were conducted for urban and rural areas, higher educated individuals were less likely to have diabetes particularly in the rural population. This finding has also been demonstrated in Bahrain [29]. It is unclear whether lifestyle characteristics that were not measured in this study, such as exercise and dietary patterns, may be driving the relationship between education and diabetes. Thus, higher educated individuals may be better aware of coronary risk factors and use more effective preventive measures.

Finally, it is important to note that the prevalence of diabetes defined through blood test in Oman is considerably higher than the self-reported prevalence. Less than half of the number of individuals with diabetes as defined by fasting blood glucose reported having diabetes. Indeed the situation is more striking in the urban area of Muscat: among the urban population, 11.1% of those who declared they did not have diabetes actually did according to blood tests; compared to only 6.2% among those outside Muscat. This finding highlights the importance of health education and frequent testing in order to prevent individuals unaware they have diabetes from developing complications associated with the disease [33], particularly in the region of Muscat.

### Limitations

This study is limited by the cross-sectional nature of the data, which does not provide any indication of the direction of effect or causality. This limitation also prevents any measure of temporal changes in prevalence of diabetes and factors associated with diabetes. Longitudinal studies would serve as a complement to the present study to determine causality and directional effect of the factors.

In addition, the blood test used to diagnose diabetes for this study did not differentiate between type 1 and type 2 diabetes. Therefore, the proportion of individuals with diabetes in the lowest age group (3.6%) is likely suffering from type 1 diabetes. The risk factors associated with the two types of diabetes are different, with a stronger genetic component for type 1, therefore the significance of the other factors in the model may be underestimated. Indeed in a sensitivity analysis in which we excluded the youngest age groups (age 20–29) from the analysis, the strength of the association between blood pressure and diabetes increased and the relationship between urbanization and diabetes remained unchanged; however the relationship between marital status and diabetes disappeared.

Finally, because this study uses secondary data collected for the purpose of the National Health Survey, the use of robust standardization methods of data collection, particularly important for measures of blood pressure and anthropomorphic indicators, were not employed. Therefore the potential for minimizing systematic error is limited.

## Conclusion

This study highlights the prevalence of diabetes and associated coronary risk factors in urban Oman. Public health policy should be directed towards this area with a focus on primary prevention of diabetes, hypertension and obesity by lifestyle interventions.

As the relatively young Omani population ages in the future, there will likely be an escalation in the prevalence of diabetes and other chronic diseases. Therefore, it is important to continually monitor the coronary risk factors in Oman, as well as to direct effective public health interventions towards the high risk groups, such as those living in the capital region, individuals with high blood pressure, and obesity.

Chronic disease research in Oman and other countries in the Gulf region would be improved if national surveys collected more detailed information on reported symptoms, dietary patterns, physical exercise, and income level. These data would enable further analysis of chronic diseases and thus help to better inform public health policy-makers. Furthermore, a specially designed survey with the objective of determining risk factors of diabetes and cardiovascular disease that incorporates robust standardization methods is needed to confirm the findings of the present study.

At this time it is vital to consider the classification of urban and rural residence in Oman, and other Middle Eastern countries, in order to improve analytical studies as well as the consistency and accuracy of data presented by international organizations.

## Competing interests

The author(s) declare that they have no competing interests.

## Authors' contributions

Siba Al-Moosa conducted the literature review, drafted sections, and assisted with statistical analysis. Sara Allin conducted the statistical analyses, drafted and edited sections. Nadia Jemiai drafted and edited sections, and assisted with statistical analysis. Jawad Al-Lawati provided expertise and oversight throughout the process, and reviewed drafts. Elias Mossialos provided expertise and oversight throughout the research process, and reviewed drafts. All authors read and approved the final manuscript.
